# Effects of Smoking on the Gut Microbiota in Individuals with Type 2 Diabetes Mellitus

**DOI:** 10.3390/nu14224800

**Published:** 2022-11-13

**Authors:** Yuriko Kondo, Yoshitaka Hashimoto, Masahide Hamaguchi, Ayumi Kaji, Ryosuke Sakai, Ryo Inoue, Saori Kashiwagi, Katsura Mizushima, Kazuhiko Uchiyama, Tomohisa Takagi, Yuji Naito, Michiaki Fukui

**Affiliations:** 1 Department of Endocrinology and Metabolism, Graduate School of Medical Science, Kyoto Prefectural University of Medicine, Kyoto 602-8566, Japan; 2 Department of Diabetes and Endocrinology, Matsushita Memorial Hospital, Moriguchi 570-8540, Japan; 3 Laboratory of Animal Science, Department of Applied Biological Sciences, Faculty of Agriculture, Setsunan University, Hirakata 573-0101, Japan; 4 Molecular Gastroenterology and Hepatology, Graduate School of Medical Science, Kyoto Prefectural University of Medicine, Kyoto 602-8566, Japan; 5 Department for Medical Innovation and Translational Medical Science, Graduate School of Medical Science, Kyoto Prefectural University of Medicine, Kyoto 602-8566, Japan; 6 Department of Human Immunology and Nutrition Science, Kyoto Prefectural University of Medicine, Kyoto 602-8566, Japan

**Keywords:** smoking, dietary intake, gut microbiota, type 2 diabetes mellitus

## Abstract

Smoking affects eating habits; however, few studies on smoking and the gut microbiota have reported the effects of diet in detail. This cross-sectional study aimed to determine the association between smoking and the gut microbiota, considering the impact of smoking on dietary intake. Dietary habits and the composition of the gut microbiota were assessed in 195 men with type 2 diabetes (164 non-current smokers and 31 current smokers) using a brief self-administered diet history questionnaire and 16S ribosomal RNA gene sequencing of fecal samples. The data were compared according to the current smoking status of the participants. Current smokers had high alcohol and sugar/sweetener intake and low fruit intake. The proportion of the *Coprococcus* genus was higher among current smokers. Multiple regression analysis adjusted for current smoking, age, exercise habits, alcohol intake, sugar and sweetener intake, and fruit intake showed that smoking was associated with the proportion of the *Coprococcus* genus. Current smoking was associated with both dietary intake and composition of the gut microbiota. Although dietary intake should be considered when investigating the association between smoking and the gut microbiota, the results suggest that the direct effect of smoking is more significant.

## 1. Introduction

Cigarette smoking increases the risk of incident diabetes mellitus and exacerbates the microvascular and macrovascular complications of diabetes mellitus [1]. Smoking has also been shown to increase insulin resistance [2]. Moreover, smoking causes systemic inflammation and oxidative stress [3], which not only reduces vascular endothelial function [4] but also directly damages pancreatic β-cell function [5].

Smoking has also been shown to affect the gut microbiota [6]. Gut microbiota affects the regulation of nutritional metabolism [7] and of the immunological and defense mechanisms in the body [8]. Gut dysbiosis participates in the development and progression of many diseases, including type 2 diabetes mellitus [9] and inflammatory bowel disease [10]. Many factors influence the gut microbiota, including diet, lifestyle, smoking, medications, and the genetic background of the host. Weight gain often occurs after smoking cessation [11], and changes in the gut microbiota have been suggested to play a role in this phenomenon [12,13]. Additionally, smoking has different effects on inflammatory bowel disease, a risk factor for Crohn’s disease, and a protective factor against ulcerative colitis, which may also be linked to the gut microbiota [14].

Furthermore, smokers tend to have unhealthy diets [15], including high intake of junk food [16] and energy-dense foods [17], and excessive alcohol consumption [18,19]. This has been attributed partly to changes in taste [20] and smell [21] caused by smoking. Studies have also been conducted on the neuronal and behavioral overlap between food addiction and nicotine in the brain reward system [22,23], as well as on the association between smoking and appetite-related hormones, such as leptin, glucagon-like peptide-1, and ghrelin [24,25].

Smoking, the gut microbiota, and dietary intake are all involved in the pathogenesis of type 2 diabetes; however, no studies have assessed the association between smoking and gut microbiota in people with type 2 diabetes. In addition, few studies on smoking and the gut microbiota have examined the effects of diet in detail. Recent studies have also revealed that current smoking plays a more important role than past smoking in affecting the gut microbiota [6]. Therefore, this study investigated the relationship between current smoking and the gut microbiota in people with type 2 diabetes mellitus, taking into account the impact of current smoking on dietary intake.

## 2. Materials and Methods

### 2.1. Study Population

This study was designed to determine the association between diabetes mellitus, the gut microbiota, and various background factors [26,27]. The participants were outpatients at Kyoto Prefectural University of Medicine (KPUM) Hospital and Kameoka Municipal Hospital, Japan. Fecal samples were collected from 522 individuals (17 patients with type 1 diabetes, 383 patients with type 2 diabetes, 8 patients with other types of diabetes, and 114 individuals without diabetes) between November 2016 and October 2018, who had not received antibiotics within the prior 3 months. Individuals without diabetes and those with diabetes other than type 2 diabetes were excluded from the study.

### 2.2. Data Collection

Subject data were collected from medical records on sex, age, blood pressure, height, body weight, body mass index (BMI), duration of diabetes, and available medications for dyslipidemia, hypertension, diabetes, and proton pump inhibitor use. In the questionnaire, participants who reported they were currently smoking even one cigarette per day were categorized as current smokers, whereas those who reported having smoked in the past and who reported never having smoked before were categorized as non-current smokers. The daily number of cigarettes smoked and the years of smoking history were also assessed. Participants who performed some type of sporting activity at least once a week were defined as regular exercisers [28].

Fasting venous blood samples were collected to measure plasma glucose, hemoglobin A1c, triglyceride, high-density lipoprotein cholesterol, uric acid, and creatinine levels. The estimated GFR (eGFR) was defined as 194 × creatine ^−1.094^ × age ^−0.287^ (mL/min/1.73 m^2^) (×0.739 for women) according to the equation presented by the Japanese Society of Nephrology) [29].

Habitual dietary intake data were assessed using a brief self-administered diet history questionnaire (BDHQ). The BDHQ can be used to estimate the dietary intake of 58 food and beverage items in the previous month. The BDHQ has been validated previously [30,31]. In the present study, we excluded patients who did not respond to the BDHQ and those who reported extremely high (>4000 kcal) or low (<600 kcal) energy intake, because of poor reliability [32]. When evaluating energy, protein, fat, and carbohydrate intake, the intake per kilogram of ideal body weight (IBW) was calculated to consider the differences in body size. IBW was calculated as 22× the square of the participant height (m^2^) [33]. Habitual alcohol intake was defined as alcohol consumption >20 g/day in the BDHQ analysis [34].

### 2.3. Gut Microbiota Data Sampling, DNA Extraction, Sequencing, and Data Analysis

Fecal samples were collected and analyzed for gut microbiota composition using previously described methods [35,36,37]. Preservation of the collected fecal samples was performed using a guanidine thiocyanate solution (Feces Collection kit; Techno Suruga Lab, Shizuoka, Japan). Genomic DNA was isolated and purified using a NucleoSpin Microbial DNA kit (Macherey-Nagel, Düren, Germany) and Agencourt AMPure XP beads (Beckman Coulter, Brea, CA, USA).

Sequencing libraries were generated by a two-step polymerase chain reaction (PCR) of the purified DNA samples and the prepared libraries of 250 paired-end sequences were sequenced using the MiSeq Reagent v3 kit and MiSeq (Illumina, San Diego, CA, USA), at Takara Bio’s Biomedical Center, as described in a previous study [37].

The DADA2 plugin of Quantitative Insights into Microbial Ecology 2 version 2019.4 was used for table generation for amplicon sequence variants (ASVs) [26] by setting and performing noise removal by DADA2 with the trimming length from the left set at 17 and from the right at 19. Both reads were truncated to <250 base pairs. Each ASV was classified using the sklearn classifier algorithm against Greengenes database version 13_8. Singletons and ASVs assigned to chloroplasts and mitochondria were deleted in this study. Phylogenetic trees were created using SATé-compatible phylogenetic arrangements [38]. Consequently, 6902 ASVs were obtained. Functional profiles from the 16S rRNA dataset were predicted using Phylogenetic Investigation of Communities by Reconstruction of Unobserved States version 2.1.4 [35], as previously described [37].

### 2.4. Statistical Analysis

Clinical characteristics, nutrient intake, food group intake, and the proportions of phyla and genera in the gut microbiota were compared between the non-current and current smoker groups using the Mann–Whitney U test. Multiplicity was adjusted by obtaining *q* values using the Benjamini-Hochberg method to compare the proportions of the gut microbiota.

Furthermore, to assess the effects of smoking on the composition of the gut microbiota, a multiple regression analysis was performed using smoking, smoking-related background factors, and dietary intake as covariates. To investigate multicollinearity, the variance inflation factor (VIF) was checked and all VIFs were confirmed to be less than 2.

Statistical significance was set at *p* < 0.05 and *q* < 0.1. JMP version 14.0 (SAS Institute Inc., Cary, NC, USA) was used for the statistical analyses.

### 2.5. Ethics

This study was approved by the Ethics Committee of KPUM (no. ERB-C-534 and no. RBMR-E-466-5) and was performed in accordance with the principles of the Declaration of Helsinki. Written informed consent was obtained from all participants at the time of enrollment in the study.

## 3. Results

### 3.1. Clinical Characteristics

**Figure 1:**
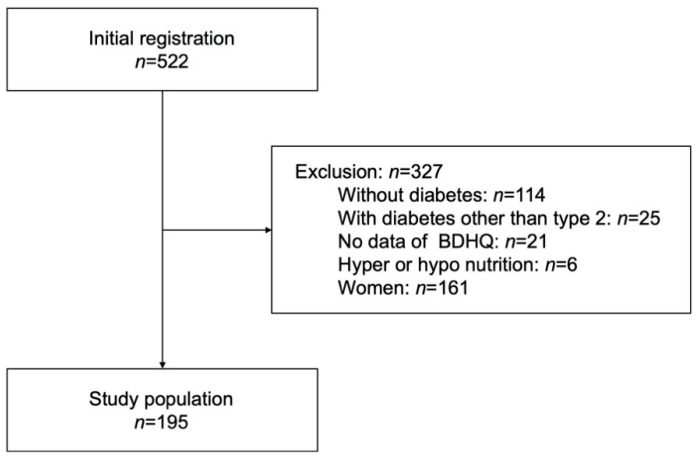
Study flow diagram of the registration of patients. BDHQ, brief self-administered diet history questionnaire.

This study analyzed the data of 195 men with type 2 diabetes mellitus (Figure 1). Women were excluded from the analysis because the number of current smokers was very low (31/195 men and 8/160 women) and to follow the methods of previous studies [6,39].

Table 1 shows the clinical characteristics of participants according to their current smoking status. Of the 195 men, 164 were non-current smokers (84.1%) and 31 were current smokers (15.9%). Comparing the two groups, the current smoker group was younger, had smoked over a longer time period, had fewer exercise habits, and had less sulfonylurea use. There were no differences in BMI, hemoglobin A1c, high-density lipoprotein cholesterol, triglycerides, uric acid, creatinine, and eGFR between the two groups.

### 3.2. Nutritional Intake and Food Group Intake

Table 2 shows the differences in nutritional intake between current and non-current smokers. Energy, protein, fat, and carbohydrate intakes did not differ between the two groups in either total intake or intake per IBW. There were also no differences in the intake of dietary fiber, sucrose, salt, and various vitamins according to the current smoking status. In contrast, current smokers consumed significantly more alcohol than did non-current smokers.

Table 3 shows food group intake compared by current smoking status. Of the 15 food groups, two food groups (“sugar/sweeteners” and “fruits”) showed differences in intake between current smokers and non-current smokers. Sugar and sweetener intake for coffee or tea and for cooking were significantly higher among the current smokers, whereas the current smokers had lower fruit intake.

### 3.3. Composition of Gut Microbiota

**Figure 2:**
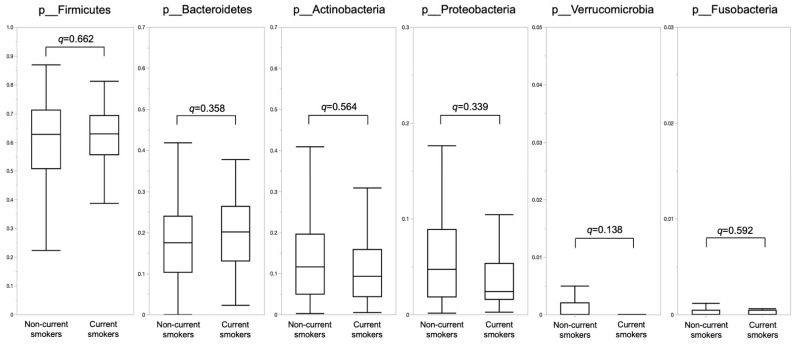
The phylum proportions by current smoking status. Differences between groups were analyzed using the Mann–Whitney U test adjusted with the Benjamini-Hochberg method.

Figure 2 shows the proportions of phyla according to the current smoking status. There was no difference between the two groups in the proportions of phyla (Table S1).

**Figure 3:**
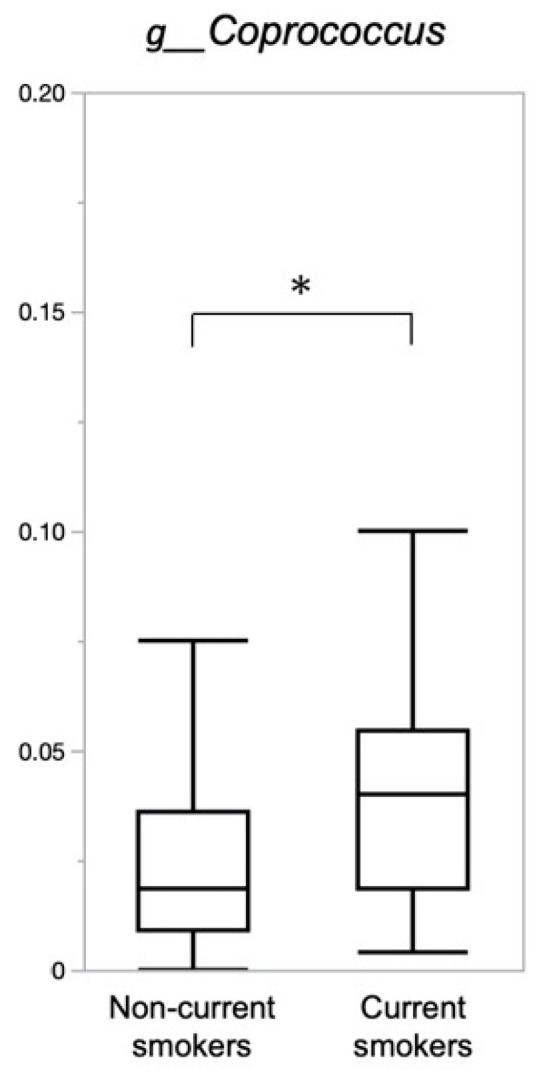
The difference in the proportion of genus *Coprococcus* by current smoking status. The difference between the groups was analyzed by the Mann–Whitney U test adjusted with the Benjamini-Hochberg method; * *q* < 0.1.

Of the highest 30 gut microbiota proportions at the genus level, the proportion of *Coprococcus* was significantly higher in current smokers than in non-current smokers (0.039 versus 0.025, *q* = 0.030) (Table S2). Figure 3 shows the difference in the proportion of *Coprococcus* according to the current smoking status.

Multiple regression analysis using current smoking status, age, exercise habits, alcohol intake, sugar and sweetener intake, and fruit intake as covariates showed that smoking was associated with the proportion of the Coprococcus genus (Table 4).

## 4. Discussion

This study of individuals with type 2 diabetes mellitus examined the association between smoking and dietary habits and between smoking and the gut microbiota. Current smokers consumed more alcohol, sugar, and sweeteners, and less fruit. We also reported that the gut microbiota of current smokers has a higher proportion of *Coprococcus* at the genus level. Current smoking was associated with a higher proportion of the *Coprococcus* genus even after adjusting for background and dietary factors related to current smoking.

Recent studies have suggested that smoking may alter the gut microbiota. Although clinical studies have been reported in healthy people [6,40], patients with inflammatory bowel disease [41,42], and patients with coronary artery disease [39], this is the first study to evaluate the association between smoking and the gut microbiota in a population of people with type 2 diabetes mellitus. Furthermore, despite many studies reporting that smoking affects eating habits [43,44], previous studies on smoking and the gut microbiota found no difference in dietary intake according to smoking status or did not assess dietary intake. A significant finding of this study is the association between current smoking and dietary intake. After considering the effects of these factors, we identified the relationship between current smoking and the gut microbiota.

A previous large cross-sectional study found no significant differences in the composition of the gut microbiota between those who had never smoked and former smokers, indicating that recovery of the gut microbiota composition to its pre-smoking status is likely to occur if smokers quit smoking [6]. Another longitudinal study on changes in the gut microbiota before and after smoking cessation showed that the effects of smoking cessation on the gut microbiota occurred quickly [12]. Similarly, the change in eating habits due to smoking cessation is also expected to occur relatively rapidly, as taste recovery was observed after two weeks [45] and weight gain appeared within three months [46] after smoking cessation. Therefore, in this study, the gut microbiota and dietary intake were compared between two groups depending on current smoking status.

The dietary habits of smokers are characterized by a low intake of vegetables and fruits, high intake of meat and alcoholic beverages, and low intake of antioxidant β-carotene and vitamin C [43,44]. The current smokers in this study also had high alcohol and low fruit intake, similar to previous findings. In contrast, the intake of vegetables and antioxidant nutrients did not differ between current and non-current smokers. This may be because of the influence of daily dietary advice and encouragement of vegetable consumption as part of the treatment of diabetes mellitus. It was also noted that the validity of the BDHQ used in this study may have been lower if only men responded without the advice from their wives [30]. Remarkably, the amounts of sugar and sweeteners used in coffee, tea, and cooking were higher among smokers in this study. Excessive intake of sucrose has been suggested to cause dysbiosis [47], and it is important to evaluate the intake of sucrose. However, the BDHQ is unreliable for measuring sucrose consumption and it is difficult to estimate the amount of sucrose consumed.

The relationship between smoking and the gut microbiota has been investigated in terms of diversity, composition, and function. Many studies have reported a reduction in the diversity of bacterial species in fecal samples from smokers [48]. Several studies have shown that smokers have a higher proportion of the Bacteroidetes phylum than nonsmokers [6,41,42]. Furthermore, there have been several reports of higher proportions of *Prevotella* in smokers [49,50,51]. The present study did not find results similar to those previously reported; however, new findings were obtained for the genus *Coprococcus*. The genus *Coprococcus* plays an important role as a protective factor against *Clostridium difficile* infection, and its proportion has been reported to decrease with strong inhibition of gastric acid secretion [52]. Smoking stimulates gastric acid secretion [53], which may increase the proportion of *Coprococcus*.

The mechanisms by which smoking affects the gut microbiota include immunosuppression, increased oxidative stress, altered gut barrier function, and changes in acid-base equilibrium [54]. Cigarette smoke contains nicotine, aldehydes, polycyclic aromatic hydrocarbons, heavy metals, toxic gases, and volatile organic compounds. The effects of each of these substances on the gut microbiota have been studied; however, the co-impact of multiple toxic substances in cigarette smoke on the gut microbiota remains unknown [55]. In addition, alcohol consumption, which was positively correlated with current smoking in this study, was reported to be associated with gut dysbiosis [56]. High fat, high sugar, and low fiber diets are also thought to cause gut dysbiosis in smokers [57]. Therefore, smoking could affect the gut microbiota not only directly through toxic substances but also indirectly through changes in dietary intake. Moreover, animal studies have indicated that a fiber-free diet further contributes to the reduction in antioxidant capacity caused by smoking [58], and smoking is expected to have a synergistic direct and indirect negative effect.

This study had several limitations. The study population of individuals with type 2 diabetes mellitus was originally prone to dysbiosis, and changes in the gut microbiota due to smoking may not have been adequately assessed. Moreover, if the direct effects of smoking on the gut microbiota are offset by indirect effects via dietary changes, they may not be detected as differences in the gut microbiota of current and non-current smokers. The quantitative impact of the number of cigarettes smoked or years of smoking was not evaluated in this study. Additionally, since this was a cross-sectional study, the causal relationship between changes in the gut microbiota, smoking, and diet is unknown.

Smoking cessation is important for people with diabetes because smoking has a significant negative influence on the pathogenesis of diabetes and its complications. Unhealthy diets consumed by patients with diabetes may have been influenced by smoking. However, many people are reluctant to quit smoking because of weight gain concerns due to smoking cessation. Weight gain associated with smoking cessation has been shown to not reduce the long-term benefit of smoking cessation in reducing cardiovascular and all-cause mortality [11,59]. More research is needed to clarify the relationship between smoking cessation and the gut microbiota, diet, and body weight because if means can be found to control weight gain with smoking cessation, more people are likely to quit smoking, and the benefits of smoking cessation would increase.

## 5. Conclusions

This study revealed that both dietary intake and the gut microbiota are associated with current smoking status in men with type 2 diabetes mellitus. However, even after adjusting for the effects of smoking on dietary intake, associations between current smoking and the gut microbiota were still observed. The results of this study suggest that although dietary intake should be considered when examining gut microbiota associations, the non-dietary effects of smoking are more significant.

## Figures and Tables

**Table 1:** Clinical characteristics of the subjects.

	Non-Current Smokers *n* = 164	Current Smokers *n* = 31	*p* Value
Age (years)	67.8 (10.9)	63.9 (9.5)	0.023
Duration of diabetes (years)	15.5 (9.7)	12.7 (8.5)	0.155
Body mass index (kg/m^2^)	23.9 (3.4)	23.5 (3.5)	0.652
Smoking amount (cigarettes/day)	18.2 (17.7)	20.3 (10.4)	0.134
Smoking duration (years)	18.9 (17.3)	43.1 (9.6)	<0.001
Exercise (−/+)	73/91	20/11	0.041
Habitual alcohol intake (−/+)	86/78	11/20	0.083
Systolic blood pressure (mmHg)	134.3 (17.5)	129.4 (21.0)	0.070
Diastolic blood pressure (mmHg)	78.9 (10.5)	80.2 (12.7)	0.799
Insulin (−/+)	127/37	21/10	0.247
Sulfonylureas (−/+)	116/48	29/2	0.008
Glinides (−/+)	153/11	30/1	0.460
Thiazolidines (−/+)	165/9	31/0	0.182
Biguanides (−/+)	100/64	20/11	0.710
Glucagon-like peptide-1 receptor agonist (−/+)	142/22	26/5	0.688
Dipeptidyl peptidase-4 inhibitors (−/+)	65/99	18/13	0.057
Sodium-glucose cotransporter inhibitors (−/+)	135/29	26/5	0.834
α-glucosidase inhibitors (−/+)	144/20	28/3	0.690
Proton pump inhibitors (−/+)	130/34	24/7	0.817
Hemoglobin A1c (mmol/mol)	56.4 (12.4)	59.8 (18.7)	0.834
Hemoglobin A1c (%)	7.3 (1.1)	7.6 (1.7)	0.834
HDL cholesterol (mmol/L)	1.5 (0.4)	1.5 (0.5)	0.942
Triglycerides (mmol/L)	1.5 (0.9)	1.7 (1.1)	0.482
Uric acid (mmol/L)	324.9 (76.1)	300.5 (82.0)	0.159
Creatinine (μmol/L)	83.6 (37.2)	80.0 (28.3)	0.474
eGFR (mL/min/1.73m^2^)	68.6 (19.7)	73.1 (22.7)	0.372

Values are presented as the mean (standard deviation) or number. HDL, high-density lipoprotein; eGFR, estimated glomerular filtration rate. Differences between the groups were analyzed using the chi-square test for categorical variables or the Mann–Whitney U test for continuous variables.

**Table 2:** Nutritional intake by current smoking status.

	Non-Current Smokers *n* = 164	Current Smokers *n* = 31	*p* Value
Total energy (kcal/day)	1874.6 (539.0)	2016.3 (645.6)	0.191
Energy (kcal/IBW/day)	30.6 (8.9)	32.6 (10.6)	0.323
Total fat (g/day)	57.8 (20.2)	58.5 (22.2)	0.986
Fat (g/IBW/day)	0.9 (0.3)	0.9 (0.4)	0.873
Fat per energy (%)	27.8 (6.4)	26.3 (5.9)	0.255
Total protein (g/day)	75.1 (25.5)	76.2 (31.0)	0.972
Protein (g/IBW/day)	1.2 (0.4)	1.2 (0.5)	0.827
Protein per energy (%)	16.1 (3.1)	15.1 (3.2)	0.092
Total carbohydrate (g/day)	237.9 (80.4)	248.1 (92.2)	0.795
Carbohydrate (g/IBW/day)	3.9 (1.3)	4.0 (1.5)	0.970
Carbohydrate per energy (%)	50.9 (9.4)	49.3 (8.3)	0.209
Dietary fiber (g/day)	12.4 (5.0)	12.4 (5.6)	0.747
Sucrose (g/day)	11.8 (8.1)	13.8 (9.0)	0.242
Salt (g/day)	11.4 (3.4)	11.9 (4.0)	0.677
Alcohol (g/day)	11.4 (23.1)	23.4 (30.0)	0.040
Vitamin A (μgRAE/day)	774.7 (542.2)	941.7 (945.0)	0.525
Vitamin B1 (mg/day)	0.8 (0.3)	0.8 (0.3)	0.989
Vitamin B2 (mg/day)	1.4 (0.5)	1.5 (0.6)	0.928
Vitamin B6 (mg/day)	1.3 (0.5)	1.4 (0.7)	0.757
Vitamin B12 (μg/day)	11.5 (6.5)	11.4 (7.1)	0.635
Vitamin C (mg/day)	120.5 (60.4)	120.6 (76.8)	0.644
Vitamin D (μg/day)	17.4 (10.5)	14.9 (9.4)	0.144
Vitamin E (mg/day)	8.0 (2.9)	7.8 (3.1)	0.593

Values are presented as the mean (standard deviation). IBW, ideal body weight. Differences between the groups were analyzed using the Mann–Whitney U test.

**Table 3:** Food group intake by current smoking status.

	Non-Current Smokers *n* = 164	Current Smokers *n* = 31	*p* Value
Cereals (g/day)	390.8 (172.5)	419.7 (191.5)	0.631
Potatoes (g/day)	35.3 (34.0)	38.2 (38.4)	0.736
Sugar and sweeteners (g/day)	4.1 (4.0)	6.5 (6.4)	0.034
Pulses (g/day)	64.0 (52.7)	57.7 (38.3)	0.816
Green and yellow vegetables (g/day)	118.7 (83.3)	113.6 (83.4)	0.649
Other vegetables (g/day)	181.0 (119.1)	198.8 (139.2)	0.670
Fruits (g/day)	124.7 (111.6)	84.6 (81.2)	0.044
Fish and shellfish (g/day)	93.6 (57.6)	85.5 (53.0)	0.457
Meat (g/day)	72.0 (45.1)	86.8 (61.7)	0.346
Eggs (g/day)	47.5 (30.9)	41.8 (26.2)	0.415
Milk (g/day)	166.0 (121.9)	181.1 (160.4)	0.879
Fat and oil (g/day)	11.3 (6.7)	12.1 (5.9)	0.267
Snacks (g/day)	40.4 (37.7)	40.2 (41.3)	0.765
Alcoholic and non-alcoholic beverages (g/day)	809.5 (465.0)	835.3 (504.1)	0.704
Seasonings (g/day)	224.9 (121.9)	253.4 (148.0)	0.287

Values are presented as the mean (standard deviation). Differences between groups were analyzed using the Mann–Whitney U test.

**Table 4:** Multiple regression analysis of the proportion of genus *Coprococcus*.

	*g__Coprococcus*	
	Standardized Coefficient *β*	*p* Value
Current smoking	0.253	<0.001
Age (years)	−0.068	0.353
Exercise	−0.050	0.495
Alcohol intake (g/day)	−0.039	0.593
Sugar and sweeteners intake (g/day)	−0.055	0.443
Fruits intake (g/day)	0.060	0.404

Smoking status was defined as non-current smoker (=0) or current smoker (=1), and exercise status was defined as non-regular exercise (=0) or regular exercise (=1).

## Data Availability

The sequence data used in this study were submitted to the Sequence Read Archive (SRA) with the accession number PRJNA766337 (available on 1 November 2021).

## Supplementary Materials

The following supporting information can be downloaded at: https://www.mdpi.com/article/10.3390/nu14224800/s1, Table S1: The proportions of phyla by current smoking status; Table S2: The proportions of genera by current smoking status.
