# Supplementary material for: Effects of Smoking on the Gut Microbiota in Individuals with Type 2 Diabetes Mellitus
Source: Nutrients. 2022 Nov 13;14(22):4800. doi: 10.3390/nu14224800 (PMC9695173; doi:10.3390/nu14224800)
Supplement: Supplementary file 1 [file nutrients-14-04800-s001.zip › nutrients-1981560-supplementary.pdf]

Supplemental Tables

Table S1. The proportions of phyla by current smoking status.

	Non-current smokers N=164	Current smokers N=31	*q* value
p__Firmicutes	0.595 (0.147)	0.609 (0.136)	0.662
p__Bacteroidetes	0.177 (0.091)	0.198 (0.089)	0.358
p__Actinobacteria	0.146 (0.125)	0.131 (0.126)	0.564
p__Proteobacteria	0.068 (0.079)	0.046 (0.049)	0.339
p__Verrucomicrobia	0.009 (0.034)	0.001 (0.003)	0.138
p__Fusobacteria	0.003 (0.012)	0.009 (0.033)	0.592

Values are presented as the mean (standard deviation). Differences between groups were analyzed using the Mann-Whitney U test adjusted with the Benjamini-Hochberg method.

Table S2. The proportions of genera by current smoking status.

	Non-current smokers N=164	Current smokers N=31	*q* value
g__Bacteroides	0.108 (0.081)	0.124 (0.094)	0.728
g__Bifidobacterium	0.096 (0.112)	0.089 (0.109)	0.810
g__Blautia	0.051 (0.037)	0.073 (0.046)	0.285
g__Ruminococcus	0.047 (0.046)	0.039 (0.046)	0.545
f__Lachnospiraceae;Other	0.044 (0.034)	0.053 (0.033)	0.555
g__Faecalibacterium	0.045 (0.041)	0.048 (0.043)	0.833
f__Enterobacteriaceae;Other	0.043 (0.072)	0.022 (0.027)	0.360
g__Collinsella	0.043 (0.041)	0.036 (0.036)	0.735
g__Roseburia	0.034 (0.040)	0.042 (0.039)	0.407
g__Streptococcus	0.036 (0.054)	0.025 (0.055)	0.455
g__Gemmiger	0.025 (0.032)	0.024 (0.025)	0.836
g__Coprococcus	0.025 (0.021)	0.039 (0.024)	0.030
f__Lachnospiraceae;g__[Ruminococcus]	0.023 (0.029)	0.027 (0.025)	0.563
g__Oscillospira	0.027 (0.027)	0.023 (0.022)	0.723
g__Prevotella	0.035 (0.066)	0.049 (0.086)	0.802
g__Lactobacillus	0.023 (0.060)	0.018 (0.039)	0.862
g__Dorea	0.017 (0.015)	0.019 (0.021)	0.849
g__Parabacteroides	0.017 (0.015)	0.013 (0.012)	0.500
f__Ruminococcaceae;Other	0.017 (0.020)	0.012 (0.017)	0.487
g__Megamonas	0.012 (0.035)	0.005 (0.016)	0.444
g__Phascolarctobacterium	0.013 (0.017)	0.015 (0.015)	0.693
f__Lachnospiraceae;g__	0.011 (0.009)	0.017 (0.017)	0.510
g__Lachnospira	0.011 (0.013)	0.014 (0.017)	0.860
o__Clostridiales;Other;Other	0.011 (0.018)	0.008 (0.009)	0.747
g__Megasphaera	0.013 (0.028)	0.011 (0.023)	0.731
f__Erysipelotrichaceae;g__[Eubacterium]	0.013 (0.022)	0.010 (0.020)	0.776

f__Ruminococcaceae;g__	0.011 (0.022)	0.009 (0.013)	0.858
g__Akkermansia	0.009 (0.034)	0.001 (0.003)	0.380
f__Christensenellaceae;g__	0.010 (0.026)	0.004 (0.007)	0.766
o__Clostridiales;f__;g__	0.008 (0.022)	0.007 (0.016)	0.855

Values are presented as the mean (standard deviation). Differences between groups were analyzed using the Mann-Whitney U test adjusted with the Benjamini-Hochberg method.
